# Pathogenesis and Diagnosis of Genitourinary Cancer

**DOI:** 10.3390/cancers13020347

**Published:** 2021-01-19

**Authors:** Charles C. Guo, Steven S. Shen, Jae Y. Ro

**Affiliations:** 1 Department of Anatomic Pathology, The University of Texas MD Anderson Cancer Center, Unit 085, 1515 Holcombe Blvd, Houston, TX 77030, USA; ccguo@mdanderson.org; 2 Department of Pathology and Genomic Medicine, Houston Methodist Hospital, Weill Medical College of Cornell University, 6565 Fannin St, Houston, TX 77030, USA; stevenshen@houstonmethodist.org

Genitourinary (GU) cancers are among the most common malignant diseases in men. In the United States, prostate, bladder, and kidney cancers are the first, fourth, and sixth most common cancers in men, respectively [1]. Recently, a number of genomic and molecular studies have provided an insight into the oncogenesis and progression of GU cancers, particularly bladder, prostate, and kidney cancers [2,3,4,5,6,7]. These comprehensive innovative analyses have led to new molecular classification based on the genomic expression profiles and the discovery of potential diagnostic and therapeutic molecular targets. The current special series includes 11 articles by international leading researchers who are involved in the investigation of several challenging issues, particularly novel potential biomarkers in GU cancers, using a variety of innovative approaches [8,9,10,11,12,13,14,15,16,17,18].

Bladder cancer (BC) is the focus of this Special Issue, with eight articles investigating various biomarkers that may have diagnostic, prognostic, and therapeutic value in BC. Small non-coding RNAs (sncRNAs) in urine have been used as noninvasive biomarkers for BC, but their use in plasma has not yet been well studied. Sabo AA et al. analyzed the expression of sncRNAs in plasma extracellular vesicles (EVs) in BC by next-generation sequencing [17]. They found that sncRNAs from plasma EVs, such as miR-4508, miR-126-3p, miR-185-5p, miR-106a-5p, and miR-10b-5p, may also be used as diagnostic biomarkers for BC. Moesin (MSN) is a molecule associated with an aggressive phenotype in malignant tumors. To evaluate the role of MSN in BC, Park et al. analyzed MSN in urine liquid-based cytology samples by proteomic analysis and immunohistochemistry, which demonstrated that upregulation of the MSN gene is associated with cancer invasion [15]. The in vitro 3D invasion study showed that inhibition of *MSN* significantly decreased invasiveness in BC cell lines, suggesting that MSN may be a potential diagnostic marker for cancer invasion in BC. DNA methylation analysis of urine samples has been used for the purpose of detection and surveillance in BC. Hentschel et al. investigated DNA methylation of urine supernatant (cell-free DNA) in comparison with full void urine and urine pellets [10]. They found that both cellular and cell-free DNA in urine can be used for methylation analysis in BC, with urine pellets being the most effective fraction. Large nested urothelial carcinoma (LNUC) is a new entity in the 2016 WHO classification of urothelial tumors. Weyerer et al. evaluated a cohort of 25 LNUC using SNaPshot analysis and immunohistochemistry [18]. Pure LNUC is characterized by a luminal-papillary phenotype, *FGFR3*-mutated, PD-L1-negative tumor, but *FGFR3* mutations are rare in mixed LNUC, suggesting a different pathway of tumor development. BC is extremely rare in patients under the age of 20 years; Im et al. studied 29 patients with young-onset bladder cancer (YBC) and found that almost all YBCs were low-grade, noninvasive, papillary tumors [12]. Whole-exome sequencing and RNA sequencing were performed in YBCs in comparison with those of adult bladder cancer (ABC) cases obtained from a public database. YBCs had a low mutation burden and less complex copy number of alterations, similar to those of the ABC group with good prognosis. None of the YBCs and ABCs with YBC-like mutations showed progression to muscle-invasive tumors, suggesting that bladder cancer with YBC-like mutations represents an indolent bladder tumor, regardless of age. Long pentraxin-3 (PTX3) is a member of the pentraxin family which regulates tumor progression through cell proliferation, angiogenesis, mobility, and immune modulation. Matarazzo et al. evaluated the differential expression of PTX3 during BC progression in cell lines, animal models, and patient samples and found that PTX3 modulation and the consequent impairment of FGF/FGR systems in BC cells have a significant impact on BC growth [8]. In addition, Ide et al. reviewed the role of steroid hormone receptors, such as androgen receptor, estrogen receptor-α, estrogen receptor-β, glucocorticoid receptor, progesterone receptor, and vitamin D receptor, in urothelial tumorigenesis [11]. De Oliveira et al. updated the status of urinary biomarkers in the diagnosis and follow-up of bladder cancer, highlighting the emerging potential role of urinary EVs in BC diagnosis and management [13].

Other GU cancers, including prostate and kidney cancers, are also investigated by various researchers in this issue. MRI imaging is a valuable tool that facilitates the selection of clinically significant or high-grade prostate cancer (PC). Roumiguié et al. investigated the respective predictive values of a biomarker-based model MDx-2 as well as clinical variables in upfront MRI image-guided prostate biopsies [16]. They found that age, prostate volume, biopsy history, MDx-2, and PI-RADS-v2 scores are significantly related to the detection of high-grade PC. MDx-2 scores and the clinical variables complement PI-RADS-v2 scores in multivariate analysis, and the two combinations outperformed PI-RADS-v2 alone. Their study supported the notion that prediction models developed for systematic prostate biopsies, including those that incorporate innovative biomarkers, need to be reassessed and eventually confirmed in the context of upfront MRI image-guided biopsies. Molecular characterization has become increasingly important in the diagnosis, treatment, and prognosis of PC. Couñago et al. evaluated the current data on molecular biomarkers for PC with an emphasis on the biomarkers and gene panels with the most robust evidence to support their application in routine clinical practice [9]. Pediatric renal tumors are a highly heterogenous group of tumors, each with its own distinct histology, molecular alterations, and clinical features. Ooms et al. discussed a pattern-based approach to the diagnosis of these kidney cancers with the aid of immunohistochemistry and molecular analysis [14].

This Special Issue presents a diverse body of research that aims to address some urgent issues in the diagnosis, prognosis, and treatment of GU cancers. A number of investigative studies evaluated new biomarkers using innovative in vivo and in intro models, which may have potential value in the management of BCs and PCs. Several researchers updated the new biomarkers in BC and PCs and proposed a new approach to the diagnosis of pediatric renal cancers. We hope this issue will provide a new perspective which will lead to an improved understanding of the complex molecular and genomic mechanisms underlying the oncogenesis and progression of GU cancers.
